# Supplementary material for: A rapid evaluation of quality of sedation and ventilation care processes for critically ill patients in Vietnam
Source: PLoS One. 2026 Apr 23;21(4):e0339157. doi: 10.1371/journal.pone.0339157 (PMC13105344; doi:10.1371/journal.pone.0339157)
Supplement: S1 File — (DOCX) [file pone.0339157.s001.docx]

# Supplementary Files

Supplementary File 1: STREAM reporting guidelines

|  | **The Standards for Rapid Evaluation and Appraisal Methods (STREAM): July 2023** | Page number | Section |
| --- | --- | --- | --- |
|  |  |  |  |
| 1. Study design | a) Define the purpose, aim or research/ evaluation questions and planned deliverables guiding the study. | 3 | Introduction |
|  | b) Provide a clear description of the intervention, programme or service being evaluated. | 3,5 | Introduction & Data collection |
|  | c) Describe any preliminary research, scoping studies or piloting methods to inform the study design. | n/a | n/a |
|  | d) Describe any theoretical frameworks or models used to guide the study design (or programme theories or theories of change in the case of rapid evaluations). | 4 | Study design |
|  | e) Indicate any relevant reporting guidelines used throughout the study. | 4 | Study design |
|  | f) If Patient and Public Involvement and Engagement (PPIE), community participation or other stakeholder advisory input was used to inform the design and implementation of the study, or to address equality, diversity and inclusion, share a description of their input. | 5 | Data collection |
|  | g) Confirm if a brief protocol or proposal was developed that outlines the research/ evaluation questions, study design, methods of data collection, PPIE involvement, analysis plans, strategy to disseminate findings including potential audience, and provision of guidance on how to use data. If possible, share links to these documents. Report any changes made in the study protocol and the reason why these changes were made. | 4 | Study design |
|  | h) Share a description of the proposed duration of the study, and if any changes occurred, confirm the actual duration of the study including the data collection and data analysis periods. | 4 | Study design |
|  | i) Provide a clear description of the sampling approach, and the groups selected for the study, and explain why these approaches were taken. Clearly state if any groups or sites were not included in the study. | 5 | Data collection |
|  | j) Adhere to good practices linked to informed consent, share a description of the process used for informed consent and recruitment of study participants. | 5 | Data collection |
| 2. Evaluation or research team | a) Provide a clear description and/or rationale of the team size (including any changes over time). | 5 | Evaluation tea |
|  | b) Describe the researcher’s/ evaluator’s relationship with (whether they have had previous engagement with the site) and in proximity to the research/ evaluation site. Including whether research/evaluation is conducted virtually or face-to-face, or whether the researcher/evaluator is based in the area of the data collection. | 5 | Evaluation tea |
|  | c) Describe the levels of experience of team members and their backgrounds (including if any team members were part of the community, patient representatives or members of the public). | 5 | Evaluation tea |
|  | d) Indicate if team members received any training in rapid research/ evaluation methods. | 5 | Evaluation tea |
|  | e) Describe the roles and responsibilities of team members in this project and why the team was designed in this way. | 5 | Evaluation tea |
|  | f) If researchers/ evaluators reflected on how their background and experiences may have affected their data collection, analysis and interpretation, please describe this process (reflexivity). | 5 | Evaluation tea |
| 3. Data collection | a) Cleary describe the data collection methods used throughout the study including any rapid methods, justify why these were selected and how they were implemented. | 3,4,5,6 |  |
|  | b) If there was any translation of materials, or if data was collected in another language, share the methods that were used to ensure that conceptual equivalence and cultural validity was achieved. | 5 | Data collection |
|  | c) Provide information on any approaches, processes or practices used to ensure quality in data collection. | 5 | Data collection |
|  | d) Provide information on any approaches, processes or practices used to ensure consistency in the methods of data collection across team members. | 5 | Data collection |
|  | e) If data collection and analysis were carried out in parallel, describe the approaches, processes or practices used to facilitate this. | 6 | Data analysis |
| 4. Data analysis | a) Clearly describe the methods that were used to analyse data. If different layers of analysis were carried out in parallel (i.e., rapid analysis and more in-depth analysis), describe the approaches, processes or practices used to facilitate this. | 6 | Data analysis |
|  | b) Provide information on any approaches, processes or practices used to ensure quality in data analysis. | 6 | Data analysis |
|  | c) Provide information on any approaches, processes or practices used to ensure consistency in the methods of data analysis across team members. | 6 | Data analysis |
|  | d) If relevant, provide a clear description of the type of data triangulation that was used and how triangulation was implemented. | 6 | Data analysis |
|  | e) Confirm if any findings were shared with stakeholders as the study was ongoing, report on what was shared, if feedback was received, and whether the feedback was used to make changes to the study design. | 6 | Data analysis |
| 5. Result interpretation | a) Report if member checking was used (checking findings with study participants). Describe the approach that was used, how participant feedback was integrated, and, if not, describe why. | 6 | Data analysis |
|  | b) Describe how the findings from the study relate to the existing published literature. | 16,17,18 | Discussion |
|  | c) If relevant to the study aims, report if there were any issues with the study design that prevented transferability or comparison to existing evidence and populations. | 18 | Limitations |
|  | d) Confirm if any implications or recommendations were made based on the findings from the study. | 16,17,18 | Discussion |
|  | e) Clearly report the limitations or gaps of the study. | 18 | Limitations |
| 6. Dissemination | a) Provide a clear description of the purpose and plan of dissemination and confirm if any changes occurred to the planned dissemination. | n/a | n/a |
|  | b) Describe whether dissemination was carried out as the study was ongoing and/or after the study ended. | n/a | n/a |
|  | c) Confirm if it is possible to access the raw data from the study on request. | 19 | Declarations |
| 7. Impact | a) If possible, report on how findings were used by the commissioners of the study and/or other stakeholders, and if they were not used as planned, share the reasons for this. | n/a | n/a |
| 8. Governance and accountability | a) Include a statement on the regulatory and/or ethical approvals that were agreed, include any cases when these may not have been required and justify why. | 5 | Ethics |
|  | b) Include a statement on the funding source. | 19 | Declarations |
|  | c) Include a statement on any conflicts of interest. | 19 | Declarations |

Supplementary File 2: Registry definitions of RASS, SAT and SBT

| Spontaneous Breathing Trial (SBT) | A trial of spontaneous breathing, defined as one of the following four;  - Trial of ‘T’ piece  - Pressure support with low support (PS of 5-8)  - CPAP ( i.e. only PEEP between 0-5)  - Automatic tube compensation with CPAP  - Not eligible for SBT |
| --- | --- |
| Spontaneous Awakening Trial (SAT) | Spontaneous awakening trial (SAT or “sedation hold”) a period during which sedating medications that are being used to treat the patient were held in order to determine whether the patient requires ongoing sedation or can be managed without sedatives. |
| RASS (target) | The target RASS is a measure of sedation, awakeness and agitation. The target RASS is the target set each day on the ward round. |
| RASS (actual) | The RASS is a measure of sedation, awakeness and agitation. The actual RASS is the measured (observed) `RASS either immediately prior to a SAT or at 0800. |

Supplementary File 3: Participant Information Sheet

## Participant Information Sheet: Observations

(For stakeholders who are involved in delivering care for patients in ICU)

**Title of the study:** Stakeholder evaluation of processes of care for critically ill patients

**Introduction**

Thank you for considering to participate in this study. The Collaboration for Research Implementation and Training in Critical CARE in Asia and Africa (CCAA) is establishing a clinician-led collaborative network to improve delivery of critical care and strengthen the local health system. To achieve this, CCAA is working with [insert name of site lead] at [insert name of site] to understand existing ICU practice to inform any future quality improvement intervention.

**Purpose of the study**

This study will explore the processes in existing care and the context in which care is delivered. This includes the factors that determine current processes of care. We therefore wish to seek the perspectives of stakeholders who are involved in delivering care for patients in ICU. As someone integral to delivering care for ICU patients, your participation will be valuable for this evaluation.

**What will participation involve?**

If you give permission for the observation, a member of the research team will observe aspects of patient care and routine processes in ICU at [insert name of hospital]. They will not record your name or participate in care or discussions. They will write down notes about their observations with your consent. This observation will be in-person and will take around 2-3 hours. Where possible, patients will be informed that observation are taking place at the time.

**Risk of Participation**

There is minimal risk for you as no intervention is planned and no sensitive issues will be discussed.

**Benefits of Participation**

The findings of this study will directly inform the design and implementation of future quality improvement interventions at [insert name of hospital]. The results from all observations will be shared back with you and the team in an effort to help identify and overcome possible barriers to improving care. We also hope enablers will encourage and accelerate shared learning and improvement in care.

**Withdrawal**
You don’t have to agree to take part. You are able to withdraw consent at any time you wish. If done prior to, during, or within 72 hours of an observation, data collected will be deleted and excluded from the study. If consent is withdrawn over 72 hours after an observation, the data provided will be retained for analysis and published in a fully anonymized form.

**Privacy and confidentiality**

All data will be stored securely on a designated shared drive storage space, which is only accessible by study staff and authorised personnel. The study will comply with the EU General Data Protection Regulations and local data protection regulations. Your de-identified data may be uploaded to data repositories or shared with other researchers in line with the MORU’s data sharing policy.

**Compensation**

There are no expenses expected to arise from participating in this study and no financial or non-financial incentives will be provided for participation.

**Publications and dissemination**

Results of this study will be published as academic publications and presented in academic conferences. Reports will be compiled in different formats to be shared with multiple groups such as ethics committees, professional collaborators, CCAA members, university researchers and other organisations working with CCAA, Wellcome, UKRI, the general public as well as yourself.

**Complaints or concerns**

In case of any concern, please contact the Principal Investigator or the local lead researcher (refer to contact details). In case you require to file a formal complaint, you can email the local ethics committee (email address and telephone number - Pending) or the Oxford Tropical Research Ethics Committee (OxTREC) (oxtrec@admin.ox.ac.uk).

Contact details

For further information, please contact:

- Principal Investigator: Dr Abi Beane and Dr Duncan Wagstaff
- Local Lead Researcher:[title, name, local phone number and email of national lead researcher]

**Data protection**

The* *relevant institution* is the data controller with respect to your personal data, and as such will determine how your personal data is used in the study. The University will process your personal data for the purpose of the research outlined above. Research is a task that is performed in the public interest. Further information about your rights with respect to your personal data is available from

## Participant Information Sheet: Focus Group Discussions

(For stakeholders, patient or family members who are involved in, or affected by, the delivery of care in ICU)

**Title of the study:** Stakeholder evaluation of processes of care for critically ill patients

**Introduction**

Thank you for considering to participate in this study. The Collaboration for Research Implementation and Training in Critical care in Asia and Africa (CCAA) is establishing a clinician-led collaborative network to improve delivery of critical care and strengthen the local health system. To achieve this, we are working with [Insert name of national site lead] at [name of the hospital] to understand existing practice and the context in which care is delivered to inform future quality improvement intervention.

**Purpose of the study**

This study will explore the processes in existing care and the context in which care is delivered. We therefore wish to seek the perspectives of stakeholders who are involved in those processes. As someone receiving care in ICU/integral to delivering care for ICU patients [delete as appropriate], your input will be valuable for this evaluation.

**What will participation involve?**

After explaining the details of the study, you will be given time to consider the information and ask questions to help you decide whether to participate. If you wish to participate in the study, the study staff will ask you to give consent.

The focus group discussion will involve asking you some questions about [insert process of interest] and writing some notes with your consent. The valuable information you provide will be kept fully anonymous. This focus group discussion will be in-person and will take around 20-30 minutes.

**Risk of Participation**

There is minimal risk for you as no intervention is planned and no sensitive issues will be discussed.

**Benefits of Participation**

The findings of this study will directly inform how to improve the quality of care at [insert name of hospital]. Results from all focus group discussions will be shared back with the local team in an effort to help identify and overcome possible barriers to improving care.

**Withdrawal**
You don’t have to agree to take part. You are able to withdraw consent at any time you wish. If done prior to, during or within 72 hours after a focus group discussion, data collected will be deleted and excluded from the study. If consent is withdrawn over 72 hours following the completion of a focus group discussion, the data provided will be retained for analysis and publication in a fully anonymised form.

**Privacy and confidentiality**

Written notes will be taken during the focus group discussion with your permission. These notes will be fully anonymised,scanned and stored securely on a project designated shared drive storage space, which is only accessible by study staff and authorised personnel. After scanning, the written notes will be shredded. The study will comply with the EU General Data Protection Regulations and local data protection regulations. Your de-identified data may be uploaded to data repositories or shared with other researchers in line with the host institutions ’s data sharing policy.

Your name will only be noted to document your verbal consent to participate in the study on a separate document scanned and stored securely and separately from focus group discussion notes.

**Compensation**

There are no expenses expected to arise from participating in this study and no financial or non-financial incentives will be provided for participation.

**Publications and dissemination**

Results of this study will be published as academic publications and presented at academic conferences. Reports will be compiled in different formats to be shared with multiple groups such as ethics committees, professional collaborators, CCAA members, university researchers and other organisations working with CCAA, Wellcome, UKRI, the general public as well as yourself.

**Complaints or concerns**

In case of any concern, please contact the Principal Investigator or the local lead researcher (refer to contact details). In case you require to file a formal complaint, you can email the local ethics committee (email address and telephone number - Pending) or the Oxford Tropical Research Ethics Committee (OxTREC) ([oxtrec@admin.ox.ac.uk](mailto:oxtrec@admin.ox.ac.uk)).

Contact details

For further information, please contact:

- Principal Investigator: Dr Abi Beane, or Dr Duncan Wagstaff
- Local Lead Researcher: [title, name, local phone number and email of national lead researcher]

**Data protection**

The University of Oxford is the data controller with respect to your personal data, and as such will determine how your personal data is used in the study. The University will process your personal data for the purpose of the research outlined above. Research is a task that is performed in the public interest. Further information about your rights with respect to your personal data is available from <http://www.admin.ox.ac.uk/councilsec/compliance/gdpr/individualrights>[**/**](http://www.admin.ox.ac.uk/councilsec/compliance/gdpr/individualrights/)

Supplementary File 4: Structured observation guide

Date: Time Start: Observer:

Site ID: End:

Team members present (note down roles, not names):

**Observation Template Sheet**

**Title of the study:** Stakeholder evaluation of processes of care for critically ill patients

| Please note down which care processes of interest are observed | |
| --- | --- |
| **1. Characteristics of the behaviour/task**   - How does [insert task/process] normally happen? - Who performs [insert task/behavior of interest]? - How often is [insert task/behaviour of interest] performed for a patient? - When is [insert task/behavior of interest] performed? - Has [insert process of interest] always been done this way? - When are decisions about [insert task/behaviour of interest] made? (eg ward round) - Who makes decisions regarding [insert process/behaviour of interest] |  |
| **Information system(s):**   - What information is needed to decide/perform [insert behaviour of interest]? - Is this information available and accessible? - How does information relevant to [insert behaviour of interest] get communicated or documented? |  |
| **2. Professional interactions**  **Communication & influence**   - What influences performing [insert task/behaviour of interest]? - Whose opinion matters in performing [insert task/process of interest]? |  |
| **Professional interactions:**  **Team Processes**   - How do team members interact and communicate when discussing or performing [insert task/behaviour of interest]? - How does [insert behaviour of interest] relate to other care processes performed by the team? - What factors are enabling or hindering performance of the [insert behaviour of interest]? - Are transitions in care relevant eg arrival to ICU/shift change handover? - What communication happens between referring clinician and ICU team? |  |
| **Patient factors**  **Patient needs:**  Did patients or relatives express what they want or expect from [insert process of interest]?  Were there any patient behaviours that helped [insert process of care] take place? Were there any patient behaviours that made it more difficult? |  |
| **Incentives & disincentives**  **Financial & non-financial**   - What financial incentives exist for patients, staff or organisations that promote existing practice? - What non-financial incentives exist? |  |
| **Any other observations related to**   - Comment on specific enablers and barriers to performance of behaviour of interest |  |

Supplementary File 5: Focus group discussion guide

**Focus Group Discussion Topic Guides**

**Title of the study:** Stakeholder evaluation of processes of care for critically ill patients

**Interviewer guidance**

Please refer to the process map and any observations made to prompt or generate questions throughout the interview when discussing [insert the process of care under evaluation]. Questions can be targeted to what has directly been observed during observations.

**Introduction and Informed Consent**

Introduce yourself

Explain the Participant Information Sheet to the participant before the interview and answer any questions they may have. Ask these questions after the explanation to confirm participation:

- **Do you agree to take part?** Commence only once the participant agrees to this.

Focus group discussons by nature are iterative and the questions evolve in the context of the conversation. Participants will take an active role in shaping the focus group discussion, so as to facilitate identification of themes specific to the particpants’ context and experience that might be overlooked using traditional interview formats. Broadly, the focus group discussions will follow the following structure for healthcare providers;

**Experiences with managing critically ill patients generally**

Could you please explain about your experience of caring for critically ill patients?

**Challenges with specific processes of care for critically ill patients**

What obstacles do you encounter when caring for critically ill patients?

Can you please provide specific examples related to the processes of care being explored? (Such as antimicrobial management, ventilation care bundle).

Can you explain why you think these challenges exist? *(consider expertise, skills, confidence, support from team members, communication (including handovers and documentation) availability of equipment, resources (including time), perceptions of priority/ importance of the process of care, and patients interactions).*

Have you experienced these challenges previously in other hospitals or departments?

Do you think others in the same role (and then in different roles to you) experience the same challenges? If different, why do you think their experience is different?

What would you recommend as the best strategies to overcome these challenges?

(How) Have you tried to overcome these challenges? What worked well and what might you do differently?

Supplementary File 6: RAP sheets

**Rapid Assessment Process (RAP) Sheet**

[Insert site name]

| **Title of the study:** Stakeholder evaluation of processes of care for critically ill patients  **Data Collection**: | |
| --- | --- |
| Mapping with staff (M) |  |
| Observations (O) |  |
| Interviews with stakeholders | Written summaries of go-along interviews (up to 20) |

**Findings**

The main findings below are to be read in conjunction with the [insert name] process map.

| **Topics** | **Main findings** |
| --- | --- |
| **Process structure**   1. *Most common process* 2. *Deviations from common process* 3. *Main handovers/ transfers?* |  |
| **History of the pathway/changes through time** |  |
| **Elements that work well** |  |
| **Areas that need to be improved**   1. Bottlenecks, contributing problems and impact 2. Drivers for problems and bottlenecks |  |
| **Types of patients receiving care** |  |
| **Plans to make changes in the near future** |  |
| **Patient needs**  *Were these met?* |  |
| **Additional comments**   1. *Differences between staff perspectives* 2. *Differences between staff perspectives and observations* 3. *Differences between observations and patient interviews* |  |

Supplementary File 7: Reflexivity statement

Structured reflexivity statement

| Study conceptualisation | How does this study address local research and policy priorities? | It studies quality of locally prioritised care processes. |
| --- | --- | --- |
|  | How were local researchers involved in study design? | CLT and YLM co-authored the published study protocol |
| Research management | How has funding been used to support the local research team(s)? | All the funding was spent to support local research team in Vietnam. |
| Data acquisition and analysis | How are research staff who conducted data collection acknowledged? | ALP collected qualitative data and is recognised as first author. Registry data collectors are also acknowledged. |
|  | How have members of the research partnership been provided with access to study data? | Collaborative zoom meetings were used to discuss emergent findings. |
|  | How were data used to develop analytical skills within the partnership? | ALP has previous qualitative research experience but has no clinical knowledge to understand all of procedures. |
| Data interpretation | How have research partners collaborated in interpreting study data? | Collaborative zoom meetings were used to discuss emergent findings. |
| Drafting and revising for intellectual content | How were research partners supported to develop writing skills? | ALP wrote first draft with subsequent discussion and suggestions from DW |
|  | How will research products be shared to address local needs? | Findings already shared locally to guide local QI |
| Authorship | How is the leadership, contribution and ownership of this work by LMIC researchers recognised within the authorship? | LMIC first authorship and numerous co-authors |
|  | How have early career researchers across the partnership been included within the authorship team? | Early career researchers first and last authors |
|  | How has gender balance been addressed within the authorship? | The research team consists of 10 females and 14 males. First author is a female |
| Training | How has the project contributed to training of LMIC researchers? | First use of rapid evaluation methods for LMIC research team |
| Infrastructure | How has the project contributed to improvements in local infrastructure? | Findings used to improve clinical processes |
| Governance | What safeguarding procedures were used to protect local study participants and researchers? | Local IRB approvals obtained. International ethical approval obtained (OxTREC). |

Supplementary File 8: Process mapping guide

**Mapping Session Guide**

**Title of the study:** Stakeholder evaluation of processes of care for critically ill patients

Research team present [Initials]:

Number of participants:

Role and gender of each participant (do not note down names) e.g. male staff nurse, female nurse in-charge.

**Introduction**

- Introduce the research team, clearly state voluntary nature of the session, that it will be video recorded, explain why and how data will be used and stored securely
- Check individual participant consent to proceed
- Ask the participants to introduce themselves (first name and role)
- Record the number of participants, their roles and gender
- Outline the purpose of the project and the mapping session, expected outcome and answer any questions the participants may have.
- Provide the participants with a large piece of paper, markers and post-it notes.

**Questions for the participants to work through as they build the map**

- - When does this process start? When does it end?
  - Who might be the key influencers, gatekeepers and decision-makers for each stage in the pathway (e.g. persons who may steer someone towards or away from health services)?
  - What parts of the process work well?
  - What parts of the process do not work well?
  - What are the barriers to the process happening as intended? This might include resource limitations, team working structures, availability (or absence of information).
  - How can we improve these?
- At what point does the process start?
- What happens next? And then? For each event described, answer:
  - What happens? Does this always happen? If not, what else might happen?
  - Where does it happen?
  - Who is involved and what is their role?
  - What decisions are made?
  - What data is recorded or gathered?
  - How much time does the event take? Are there any delays?
- At what point does the process end?
- Are there any strengths or positive aspects to this pathway? Anything that is done well?
- Any suggestions to improve the pathway?
- Has this pathway always been like this? Are there any plans to make changes to the process in the near future?
- What disincentives exist to changing the way this process happens?

**Conclusion**

- Summarise the main events in the process to check understanding. Clarify any uncertainties.
- Ask participants if there is anything else they would like to say about the process

Thank the participants for their time and input, and explain how the results will be shared with them.

Supplementary File 9: MUSIQ scores

|  | Baseline | Follow-up | Change | Mean |
| --- | --- | --- | --- | --- |
| ICU1 | 142 | 125 | -17 | 133.5 |
| ICU2 | 150 | 150 | 0 | 150 |
| ICU3 | 119 | 88 | -31 | 103.5 |

Interpretation of MUSIQ scores

Total Score

168 Highest possible MUSIQ score

120-168 Project has a reasonable chance of success

80-119 Project could be successful, but possible contextual barriers

50-79 Project has serious contextual issues and is not set up for success

25-49 Project should not continue as is; consider deploying resources to other improvement activities

24 Lowest Possible MUSIQ Score when all questions are answered

1 Lowest Possible MUSIQ Score (questions recorded as "don't know" or "N/A")
