# Supplementary material for: A rapid evaluation of quality of sedation and ventilation care processes for critically ill patients in Vietnam
Source: PLoS One. 2026 Apr 23;21(4):e0339157. doi: 10.1371/journal.pone.0339157 (PMC13105344; doi:10.1371/journal.pone.0339157)
Supplement: Attachment — Submitted filename: Response to reviewers.docx [file pone.0339157.s004.docx]

*Dear editors,*

*Many thanks for your patience – translating the original Vietnamese documents took us some time.*

*Please see our responses below in italics.*

1)Please upload a copy of the original ethics approval letter from the hospital IRBs (refs 3355/QD-BVBND & 4365/QD-BVBND), as required by question 1 of the human subject research checklist. If the original letter is not in English, please also provide an English translated version in the supporting file. Please note that if this documentation is not included when your manuscript is resubmitted, it may be rejected.

*We have uploaded English and Vietnamese versions of the two IRBs (refs 3355/QD-BVBND & 4365/QD-BVBND).*

2)We noted a different IRB approval number (558-20) in your online submission form which does not match with the approval document (525-21). To proceed with the manuscript, we request you to provide the correct IRB document or update the correct IRB approval number in the online submission form. Please note that some fields in the submission form may reset with this change, so please go through your submission in full to ensure that all information is accurate and complete when resubmitting your manuscript.

*We have uploaded the ethics approval from OxTREC for 03NVb (re f558-20).*

3)In the ethics statement in the Methods, you have specified that verbal consent was obtained. Please provide additional details regarding how this consent was documented and witnessed, and state whether this was approved by the IRB.

*We collected written consent for participation from interview and Focus Group Discussion participants, and have uploaded Participant Information Sheets (PIS) and Consent Forms for both studies. For the observations, verbal consent was sought (but not documented) in accordance with IRB approval.*

Kind regards,

The authors
