# Supplementary material for: A rapid evaluation of quality of sedation and ventilation care processes for critically ill patients in Vietnam
Source: PLoS One. 2026 Apr 23;21(4):e0339157. doi: 10.1371/journal.pone.0339157 (PMC13105344; doi:10.1371/journal.pone.0339157)
Supplement: Attachment — Submitted filename: Commentary Luu PlosOne 032725.docx [file pone.0339157.s003.docx]

Pulmonary Disease and Critical Care Medicine

Henry Ford Health System

2799 West Grand Boulevard

Detroit, MI, USA 48202

March 27, 2025

To The Editors:

Thank you very much for allowing me to review the article by Luu and coworkers entitled “A rapid evaluation of quality of sedation and ventilation care processes for critically ill patients in Vietnam”. I found this paper to be interesting and informative. The authors used a variety of methods to assess practices in three intensive care units in Vietnam regarding ventilator liberation. I found the methods used to assess practices to be excellent. Resource limitations have a significant impact on ventilator liberation practices in Vietnam. This work not only highlighted the fact that limitations exist, but provided detailed assessments that will allow physicians in this region to improve medical care.

If I were to make a critical comment about this work, it would be that there should be references to clinical practice standards and guidelines in the domain of ventilator liberation. Clearly, such guidelines have been developed only for research-rich environments and could not be applied to intensive care units in Vietnam without modification. However, tools are available to modify existing clinical practice guidelines to regions and nations where there are differences in population demographics and economic situations from those regions for which the original guidelines were developed. This additional commentary could provide a template for future improvements in the quality of care in Vietnam.

Sincerely,

Daniel R. Ouellette MD

Chief, Pulmonary and Critical Care Medicine

Henry Ford Health System
