# Supplementary material for: A rapid evaluation of quality of sedation and ventilation care processes for critically ill patients in Vietnam
Source: PLoS One. 2026 Apr 23;21(4):e0339157. doi: 10.1371/journal.pone.0339157 (PMC13105344; doi:10.1371/journal.pone.0339157)
Supplement: Attachment — Submitted filename: Response_to_reviewers_auresp_1.docx [file pone.0339157.s005.docx]

Rebuttal letter

Many thanks for the opportunity to revise and improve our manuscript.

Our point by point responses are in italics below.

Specific reviewer comments:

1. Please ensure that your manuscript meets PLOS ONE's style requirements, including those for file naming. The PLOS ONE style templates can be found at

https://journals.plos.org/plosone/s/file?id=wjVg/PLOSOne_formatting_sample_main_body.pdf

and

https://journals.plos.org/plosone/s/file?id=ba62/PLOSOne_formatting_sample_title_authors_affiliations.pdf

*We have amended the manuscript to meets PLOS ONE's style requirements as suggested.*

2. Please include a complete copy of PLOS’ questionnaire on inclusivity in

global research in your revised manuscript. Our policy for research in this area

aims to improve transparency in the reporting of research performed outside of

researchers’ own country or community. The policy applies to researchers who

have travelled to a different country to conduct research, research with

Indigenous populations or their lands, and research on cultural artefacts. The

questionnaire can also be requested at the journal’s discretion for any other

submissions, even if these conditions are not met. Please find more information

on the policy and a link to download a blank copy of the questionnaire here:

https://journals.plos.org/plosone/s/best-practices-in-research-reporting. Please

upload a completed version of your questionnaire as Supporting Information

when you resubmit your manuscript.

*We have included a complete copy of PLOS’ questionnaire on inclusivity in global research.*

3. Thank you for stating the following financial disclosure:

“This research was funded by Wellcome [(224048/Z/21/Z), (107367/Z/15/Z),

(089276/B/09/7), (217650/Z/19/Z)].”

Please state what role the funders took in the study. If the funders had no role,

please state: "The funders had no role in study design, data collection and

analysis, decision to publish, or preparation of the manuscript."

4 of 9If this statement is not correct you must amend it as needed.

Please include this amended Role of Funder statement in your cover letter; we will change the online submission form on your behalf.

*We have amended the wording to the funding declaration as suggested*

4. In the online submission form, you indicated that [All data are available upon

request.].

All PLOS journals now require all data underlying the findings described in their

manuscript to be freely available to other researchers, either 1. In a public

repository, 2. Within the manuscript itself, or 3. Uploaded as supplementary

information.

This policy applies to all data except where public deposition would breach

compliance with the protocol approved by your research ethics board. If your

data cannot be made publicly available for ethical or legal reasons (e.g., public

availability would compromise patient privacy), please explain your reasons on

resubmission and your exemption request will be escalated for approval.

*We have amended the declaration to clarify that all data are available through managed access via the OUCRU Data Access Committee (and provided the website for how to do this). This follows the standard policy of OUCRU, Oxford University and Welcome, to avoid exploitation of LMIC researchers and institutions and enable data compliance with both UK and Vietnamese law.*

5. When completing the data availability statement of the submission form, you

indicated that you will make your data available on acceptance. We strongly

recommend all authors decide on a data sharing plan before acceptance, as the

process can be lengthy and hold up publication timelines. Please note that,

though access restrictions are acceptable now, your entire data will need to be

made freely accessible if your manuscript is accepted for publication. This policy

applies to all data except where public deposition would breach compliance with

the protocol approved by your research ethics board. If you are unable to adhere

to our open data policy, please kindly revise your statement to explain your

reasoning and we will seek the editor's input on an exemption. Please be assured

that, once you have provided your new statement, the assessment of your

exemption will not hold up the peer review process.

*Please see response to 4. above*

6. Please amend the manuscript submission data (via Edit Submission) to include

authors Vu Dinh Phu, Doan Bui Xuan Thy, and Pham Ngoc Thach.

*We have amended the manuscript submission data (via Edit Submission) to include authors Vu Dinh Phu, Doan Bui Xuan Thy, and Pham Ngoc Thach.*

7. We notice that your supplementary files are included in the manuscript file.

Please remove them and upload them with the file type 'Supporting Information'.

Please ensure that each Supporting Information file has a legend listed in the

manuscript after the references list.

*We have uploaded supplementary files as a separate document and included a legend in the manuscript after the references*

8. Please review your reference list to ensure that it is complete and correct. If

you have cited papers that have been retracted, please include the rationale for

doing so in the manuscript text, or remove these references and replace them

with relevant current references. Any changes to the reference list should be

mentioned in the rebuttal letter that accompanies your revised manuscript. If you

need to cite a retracted article, indicate the article’s retracted status in the

References list and also include a citation and full reference for the retraction

notice.

*Our reference list is now complete and correct.*

Additional Editor Comments:

The study evaluated ventilator liberation practices in three Vietnamese ICUs

using robust methodologies despite resource constraints. While the work

highlights significant limitations impacting care, it provides a valuable

foundation for improving medical practices in the region. However, Reviewer 3

noted that the paper would benefit from referencing clinical practice guidelines

and discussing how these guidelines could be adapted to Vietnam's unique

context, offering a framework for future enhancements in care quality. Please

address the comments below and resubmit your manuscript for further

consideration.

*As suggested, we have added a sentence in the Discussion pointing out tools to adapt clinical practice guidelines between settings. We feel this completes the existing extensive discussion of the specific aspects of Vietnamese context which necessitate adaptation of international guidelines.*

*As suggested, we have added a sentence to stress the limitations on generalisability due to sampling issues*

Kind regards,

The authors.
